# Fluralaner activity against life stages of ticks using *Rhipicephalus sanguineus* and *Ornithodoros moubata* IN *in vitro* contact and feeding assays

**DOI:** 10.1186/s13071-015-0704-x

**Published:** 2015-02-08

**Authors:** Heike Williams, Hartmut Zoller, Rainer KA Roepke, Eva Zschiesche, Anja R Heckeroth

**Affiliations:** MSD Animal Health Innovation GmbH, Research Antiparasitics, Zur Propstei, 55270 Schwabenheim, Germany

**Keywords:** *Rhipicephalus sanguineus*, *Ornithodoros moubata*, Fluralaner, Acaricidal activity, Tick life stages

## Abstract

**Background:**

Fluralaner is a novel isoxazoline eliciting both acaricidal and insecticidal activity through potent blockage of GABA- and glutamate-gated chloride channels. The aim of the study was to investigate the susceptibility of juvenile stages of common tick species exposed to fluralaner through either contact (*Rhipicephalus sanguineus*) or contact and feeding routes (*Ornithodoros moubata*).

**Methods:**

Fluralaner acaricidal activity through both contact and feeding exposure was measured *in vitro* using two separate testing protocols. Acaricidal contact activity against *Rhipicephalus sanguineus* life stages was assessed using three minute immersion in fluralaner concentrations between 50 and 0.05 μg/mL (larvae) or between 1000 and 0.2 μg/mL (nymphs and adults). Contact and feeding activity against *Ornithodoros moubata* nymphs was assessed using fluralaner concentrations between 1000 to 10^−4^ μg/mL (contact test) and 0.1 to 10^−10^ μg/mL (feeding test). Activity was assessed 48 hours after exposure and all tests included vehicle and untreated negative control groups.

**Results:**

Fluralaner lethal concentrations (LC_50_, LC_90/95_) were defined as concentrations with either 50%, 90% or 95% killing effect in the tested sample population. After *contact* exposure of *R. sanguineus* life stages lethal concentrations were (μg/mL): larvae - LC_50_ 0.7, LC_90_ 2.4; nymphs - LC_50_ 1.4, LC_90_ 2.6; and adults - LC_50_ 278, LC_90_ 1973. After exposure of *O. moubata* nymphs to fluralaner lethal concentrations were (μg/mL): contact exposure - LC_50_ 720_,_ LC_95_ 1133; and feeding exposure- LC_50_ 0.00007, LC_95_ 0.09.

**Conclusions:**

Fluralaner demonstrates potent *in vitro* acaricidal activity against all life stages of the brown dog tick, *R.sanguineus*. The testing of fluralaner contact and feeding routes using *O. moubata* nymphs demonstrates a high acaricidal activity in both exposure routes.

## Background

Fluralaner is a new molecular entity of the isoxazoline class and provides potent acaricidal and insecticidal activity through a dual mechanism of binding to both neuronal GABA and glutamate channels in susceptible invertebrates [1,2]. The favorable properties of fluralaner led to the development of a novel veterinary parasiticide (Bravecto™, MSD Animal Health), whose remarkably long duration of activity against tick and flea infestations after the oral administration to dogs was confirmed in clinical field studies in different geographic regions of Europe and the USA [3,4]. Only a short period of time is needed to effectively kill fleas (8 hours) and ticks (12 hours) on orally treated dogs [5,6]. This rapid speed of kill lasts for a 12-week period, a benefit for dog owners with respect to the disruption of the flea life cycle [5,7], treatment of flea allergy dermatitis (FAD) [3-5] and reducing the risk of pathogen transmission [6].

Due to its selectivity to arthropods, fluralaner has a very favorable safety profile in dogs [8] including MDR1(-/-) Collies [9]. It was shown that concurrent treatments of oral fluralaner with milbemycin oxime – praziquantel (Milbemax™, Novartis Animal Health) [10] or deltamethrin (Scalibor™ protectorband, MSD Animal Health) [11] is also well tolerated in dogs.

While fluralaner’s effects on different flea life cycle stages have already been described [7], more information regarding the susceptibility of juvenile ticks to fluralaner is desirable. The studies discussed in this paper were designed to provide quantitative *in vitro* data on the susceptibility of common tick species exposed to fluralaner through both contact and feeding routes.

## Methods

All test procedures were conducted on three replicates per species and life stage (where appropriate) along with a vehicle control (solvent concentration equivalent to that of the highest fluralaner concentration test solution) as well as an untreated negative control.

### Tick contact exposure of *R. sanguineus* life stages

A fluralaner stock solution (20 mg/mL) was diluted with deionized water to a fluralaner concentration of 1000 μg/mL and then further diluted with sufficient volume of deionized water to obtain the test concentrations between 50 and 0.05 μg/mL for larvae (i.e. 50, 25, 12.5, 6.2, 3.1, 1.5, 0.78, 0.39, 0.2, 0.1 and 0.05 μg/mL), and between 1000 and 0.2 μg/mL for nymphs and adults (i.e. 1000, 500, 100, 50, 25, 12.5, 6.2, 3.1, 1.5, 0.78, 0.39 and 0.2 μg/mL). A sufficient number of *R. sanguineus* larvae (i.e. 88-390 per replicate) or nymphs (i.e. 35-115 per replicate) were placed with a brush between two filter papers in a Petri dish and 5 mL of either test or vehicle solution was added to immerse the larvae and nymphs while another group remained untreated. Larvae were immersed in fluralaner concentrations between 50 and 0.05 μg/mL, while nymphs were immersed in fluralaner concentrations between 1000 and 0.2 μg/mL. After three minutes, the solution was poured off and the filter papers were unfolded and placed with the exterior side on a paper towel to remove excess solution. All ticks were then stripped onto a new filter paper that was folded into a sachet and sealed. Sachets were incubated at approximately 22°C and 90% relative humidity (RH) for 48 hours. The sachets were then opened and transferred to a heated plate for counting the number of live and dead larvae and nymphs.

Adult ticks were tested with fluralaner concentrations between 1000 and 0.2 μg/mL. Twenty unfed adult ticks were immersed in 5 mL of either test or negative control solution in an Erlenmeyer flask for three minutes while another group remained untreated. Then ticks were strained from the flask contents, dried on a paper towel, transferred to dry filter paper in a Petri dish and covered. Petri dishes were then incubated as for larvae and nymphs, followed by counting the number of live and dead adults on a heated plate.

### Tick contact exposure of *O. moubata*

A fluralaner stock solution (20 mg/mL) was diluted with deionized water to a test concentration of 1000 μg/mL and a series of 1:10 dilutions with deionized water were prepared to obtain fluralaner test concentrations between 1000 and 10^−4^ μg/mL (i.e. 1000, 100, 10, 1, 0.1, 0.01, 0.001 and 10^−4^ μg/mL).

Twenty unfed *O. moubata* nymphs were immersed in 5 mL of either test or a vehicle solution in an Erlenmeyer flask for five minutes or nymphs remained untreated. Ticks were then strained, dried on a paper towel, transferred into a Petri dish lined with a dry filter paper and covered. The Petri dish was incubated at 20°C and 95% RH for 48 hours. Thereafter, the number of live and dead ticks was counted on a heated plate.

### Tick membrane feeding exposure of *O. moubata*

Defibrinated sheep blood was added to a fluralaner stock solution (50 mg/mL) to produce a 1000 μg/mL fluralaner preparation that was then further diluted in series of 1:10 with sheep blood to obtain test concentrations between 0.1 and 10^−10^ μg/mL (i.e. 0.1, 0.01, 0.001, 10^−4^, 10^−5^, 10^−6^, 10^−7^, 10^−8^, 10^−9^ and 10^−10^ μg/mL).

Twenty *O. moubata* nymphs were fed using artificial membranes with either a test or vehicle control preparation or an untreated control (feeding on blood only) until engorgement. Ticks were counted and placed into a plastic vial that was then closed with a stretched membrane and placed upside down on a glass dish containing 2 mL warmed test or vehicle preparation to permit feeding. Fully engorged ticks were transferred to dry filter paper in a Petri dish and covered. Ticks were incubated at 22°C and 90% RH for 48 hours, then the dishes were opened and numbers of live and dead ticks counted on a heated plate.

#### Statistical analysis

The corrected percentages of mortality were calculated for all test methods, fluralaner concentrations and for each species and life stage (where appropriate) using Schneider-Orelli’s formula:

$$ \mathrm{Corrected}\kern0.5em \mathrm{M}\mathrm{ortality}\kern0.5em \left(\%\right)\kern0.5em =\kern0.5em \left(\mathrm{M}\kern0.5em \mathrm{in}\kern0.5em \mathrm{treated}\kern0.5em \mathrm{plot}\kern0.5em \hbox{--} \kern0.5em \mathrm{M}\kern0.5em \mathrm{in}\kern0.5em \mathrm{control}\kern0.5em \mathrm{plot}\right)/\left(100\kern0.5em \hbox{--} \kern0.5em \mathrm{M}\kern0.5em \mathrm{in}\kern0.5em \mathrm{control}\kern0.5em \mathrm{plot}\right)\kern0.5em \times \kern0.5em 100, $$

where M was the mortality (%) for each tick stage/species per concentration tested and included either ticks from all replicates of the respective test preparation (M in treated plot) or control preparation (M in control plot, i.e. the arithmetic mean mortality obtained from vehicle and untreated control). M was calculated using the following formula:

$$ \mathrm{M}\kern0.5em \left(\%\right)\kern0.5em =\kern0.5em \mathrm{arithmetic}\kern0.5em \mathrm{mean}\kern0.5em \mathrm{of}\kern0.5em \mathrm{dead}\kern0.5em \mathrm{ticks}\kern0.5em \mathrm{per}\kern0.5em \mathrm{preparation}/\mathrm{arithmetic}\kern0.5em \mathrm{mean}\kern0.5em \mathrm{of}\kern0.5em \mathrm{ticks}\kern0.5em \mathrm{used}\kern0.5em \mathrm{per}\kern0.5em \mathrm{preparation}. $$

The corrected mortality results were then used to calculate lethal concentrations LC_50_ and LC_90_ or LC_95,_ defined as concentrations with either 50%, 90% or 95% killing effect in the tested sample population. Lethal fluralaner concentrations for each exposure method were calculated using probit analysis (SAS®, release 9.2).

## Results

### Tick contact exposure of *R. sanguineus* life stages

The arithmetic mean mortality obtained from vehicle control and untreated control was 3.1% (larvae), 0.7% (nymphs) and 0% (adults). The corrected *R. sanguineus* life stage mortality (Table 1) at 48 hours after contact exposure to fluralaner were used to calculate predicted LC_50_ and LC_90_ fluralaner concentrations (Table 2). After fluralaner contact exposure of *R. sanguineus* LC_90_ results were 2.41 μg/mL (larvae), 2.61 μg/mL (nymphs) and 1973 μg/mL (adults).

**Table 1:** ***R. sanguineus*** **- mortality of different tick life stages after contact exposure to fluralaner**

**Fluralaner (μg/mL)**	**Mortality (%)** ^**a**^		
	**Larvae**	**Nymphs**	**Adults**
1000	NT	100	88.5
500	NT	100	58.5
100	NT	100	28.5
50	100	100	6.5
25	100	100	3.5
12.5	100	100	0
6.2	100	100	1.5
3.1	98.9	97.0	3.5
1.5	91.7	54.9	0
0.78	31.0	4.1	0
0.39	9.1	1.1	0
0.20	4.6	0.8	0
0.10	4.7	NT	NT
0.05	3.1	NT	NT

^a^The mortality results were corrected with the mortality in controls (i.e. larvae 3.1%, nymphs 0.7%, adults 0%) using Schneider-Orelli’s formula.

NT: not tested.

**Table 2:** **Lethal concentrations (LC) of fluralaner for different tick life stages after contact exposure**

***R. sanguineus***	**Fluralaner (μg/mL)**	
	**LC50**	**LC90**
Larvae	0.70	2.41
Nymphs	1.35	2.61
Adults	278.04	1973.0

### Tick contact and membrane feeding exposure of *O. moubata*

The arithmetic mean mortality obtained from vehicle control and untreated control was 0% after contact exposure and 2.5% after feeding exposure. The corrected *O. moubata* nymph mortality after both contact and membrane feeding exposure to fluralaner are presented (Table 3) and predicted LC_50_ and LC_95_ fluralaner concentrations were calculated (Table 4). After fluralaner contact and feeding exposure of *O. moubata* LC_95_ results were 1133 μg/mL (contact) and 0.09104 μg/mL (feeding).

**Table 3:** ***O. moubata*** **- mortality of nymphs after contact or feeding exposure to fluralaner**

**Fluralaner (μg/mL)**	**Mortality (%)** ^**a**^	
	**Via contact**	**Via feeding**
1000	88.5	NT
100	0	NT
10	0	NT
1	0	NT
0.1	0	100
0.01	0	100
0.001	0	83.0
10^−4^	0	14.4
10^−5^	NT	14.4
10^−6^	NT	9.2
10^−7^	NT	16.4
10^−8^	NT	7.7
10^−9^	NT	0
10^−10^	NT	0

^a^The mortality results were corrected with the mortality in controls (i.e. contact exposure 0%, feeding exposure 2.5%) using Schneider-Orelli’s formula.

NT: not tested.

**Table 4:** **Lethal concentrations (LC) of fluralaner for ticks after contact or feeding exposure**

***O. moubata***	**Fluralaner (μg/mL)**		
	**Exposure method**	**LC50**	**LC95**
Nymphs	Contact	719.5	1133.0
	Feeding	0.00007	0.09104

## Discussion

The results show that fluralaner has potent acaricidal activity against two different tick genera that is observed in both of the *in vitro* contact and feeding exposure routes tested.

Contact exposure via immersion of three life stages (larvae, nymphs and adults) of *R. sanguineus*, all of which feed on dogs, shows that juvenile life stages are more susceptible to contact fluralaner exposure than adults, and among the juvenile stages larvae are slightly more sensitive than nymphs (Table 1). Comparison of the LC_90_ results shows that larvae and nymphs are about 700-fold more sensitive to the acaricidal activity of fluralaner than the adults. The greater acaricidal activity against juvenile ticks compared with adult ticks offers additional protection to dogs and their owners, because fluralaner treated animals that are free of visible adult ticks are even more likely to be free of smaller, and therefore more difficult to spot, juvenile ticks. The prominent efficacy demonstrated for fluralaner (Bravecto™) in a clinical field study supports this conclusion as some juvenile ticks (*Ixodes* spp.) were present on dogs when included into the study [3], indicating the possibility that dogs were exposed to larval and nymph infestations also in the further course of the study.

Comparison of the LC values between both exposure routes tested shows that fluralaner’s acaricidal potency is greater following feeding exposure than following contact exposure. This result supports that fluralaner can be highly effective for systemic administration to dogs for ectoparasite control.

*In vitro* feeding of argasid (soft) ticks, e.g. *Ornithodoros* spp., on blood through ready to use artificial membranes is a long-established method [12-14] with the advantage of ticks completing their blood meal within a few hours [14], and it is an established model at the authors’ laboratory. The method has also been adapted to several ixodid (hard) tick species [14-16] including the brown dog tick, *R. sanguineus* [17]. The drawback, however, is that the longer feeding duration until repletion of hard ticks is a challenge [14] and increases the complexity of the assay compared to soft tick *in vitro* feeding. One of the limiting factors of the *in vitro* feeding of hard ticks seems to be the length of the tick’s mouthparts [17]. The attempt to feed larvae of *R. sanguineus* on bovine blood through a silicone membrane failed because larvae did not attach [17]. Taking this into consideration, *R. sanguineus* ticks were evaluated using contact exposure tests which allowed for a direct comparison of the susceptibility of all three life stages to fluralaner.

After contact exposure, *O. moubata* exhibits similar susceptibility to fluralaner as adult *R. sanguineus* while juvenile *R. sanguineus* are even more susceptible. Thus, *O. moubata* can serve as a surrogate for *R. sanguineus* in the assessment of sensitivity to fluralaner after feeding exposure.

After feeding exposure to fluralaner, LC_95_ for *O. moubata* is extremely low (<0.1 μg/mL), far lower (around 12,000-fold) than the contact LC_95_ suggesting that fluralaner also has much greater acaricidal potency against all stages of *R. sanguineus* through feeding exposure rather than through contact exposure. The field study results [3] on client-owned dogs using oral administration of fluralaner (Bravecto™) strongly support this observation.

## Conclusions

Fluralaner demonstrates potent acaricidal *in vitro* activity against all life stages of *R. sanguineus*. The investigation of contact as well as feeding routes using *O. moubata* nymphs underlines the high acaricidal activity of fluralaner via both exposure routes. Given the fact that juvenile tick stages are even more susceptible to fluralaner than adult ticks, this molecule offers the opportunity to even more effectively control ticks across their lifecycle.
